# Interactions Between Nucleosomes: From Atomistic Simulation to Polymer Model

**DOI:** 10.3389/fmolb.2021.624679

**Published:** 2021-04-12

**Authors:** Chengwei Zhang, Jing Huang

**Affiliations:** ^1^ College of Life Sciences, Zhejiang University, Hangzhou, China; ^2^ Key Laboratory of Structural Biology of Zhejiang Province, School of Life Sciences, Westlake University, Hangzhou, China; ^3^ Westlake Laboratory of Life Sciences and Biomedicine, Hangzhou, China; ^4^ Institute of Biology, Westlake Institute for Advanced Study, Hangzhou, China

**Keywords:** chromatin, nucleosome, molecular dynamics simulation, umbrella sampling, potential of mean force, coarse-grain

## Abstract

The organization of genomes in space and time dimension plays an important role in gene expression and regulation. Chromatin folding occurs in a dynamic, structured way that is subject to biophysical rules and biological processes. Nucleosomes are the basic unit of chromatin in living cells, and here we report on the effective interactions between two nucleosomes in physiological conditions using explicit-solvent all-atom simulations. Free energy landscapes derived from umbrella sampling simulations agree well with recent experimental and simulation results. Our simulations reveal the atomistic details of the interactions between nucleosomes in solution and can be used for constructing the coarse-grained model for chromatin in a bottom-up manner.

## Introduction

Chromatin is highly compacted and condensed into the small space of the nucleus (Goloborodko et al., 2016b; Finn and Misteli, 2019). Human diploid genome contains about six billion DNA base pairs, and it will be approximately 2 m long if fully extended in a double helix (Fraser et al., 2015; Dans et al., 2016; Saurabh et al., 2016). In contrast, the size of the nucleus is only a few micrometers, and much remains to be understood that how chromatin fit into the nucleus in such a compacted and condensed way. The organization and dynamics of chromatin in the nucleus are found to be neither random nor stochastic, instead it is well defined and regulates the gene expression intricately.

The organization and folding processes of chromatin can be mainly divided into four layers: 1) The antiparallel double helical and right-handed B-DNA structure with each base pair rising up about 3.4 Å along the helical axis, which makes DNA very stable while it provides potential for the binding of proteins. 2) About 146 base pairs wrap around a histone octamer (also called histone core), including two copies each of the four histone core proteins (H2A, H2B, H3, and H4) in a left-handed superhelix, which constitute a nucleosome. Nucleosomes are the basic units of chromatin and provide controlled accessibility for DNA-binding proteins such as transcription machines and structural maintenance of chromosome (SMC) complexes. The height of a nucleosome is about 55 Å, so the formation of nucleosome compacts DNA by about 9 times as 146 base pairs × 3.4Å ÷ 55Å ≈ 9. Even though the DNA sequences binding to histone core are nonspecific, the binding affinity between some DNA sequences and histone core are higher than others (Field et al., 2008; Teif et al., 2012; Teif and Clarkson, 2019). About 75–90% of the genome are organized in the form of nucleosomes (Field et al., 2008). 3) Under the view of electron microscopes, chromatin appears as “beads on a string,” in which beads correspond to nucleosomes and the string between beads corresponds to the double helical DNA called linker. The string-like chromatin is shaped by loops (Alipour and Marko, 2012; Rao et al., 2014; Goloborodko et al., 2016a, Goloborodko et al., 2016b), topologically associating domains (TADs) (Dixon et al., 2012; Schwarzer et al., 2017; Krietenstein et al., 2020), and A/B compartments (Dekker et al., 2002; Zhao et al., 2006; Lieberman-aiden et al., 2009; Dekker et al., 2013). Because chromatins are fluid and dynamic, it is appropriate to use structural ensembles to describe the chromatin structure. While TADs and compartments fluctuate at a single-cell level, population-averaged TADs and compartments are tissue specific, meaning that their patterns are similar and conserved in one cell line and have high variability across different cell lines (Cheng et al., 2020; Contessoto et al., 2021). The variability of the organization and dynamics in this layer suggests direct impacts on the expression and regulation of genes (Finn and Misteli, 2019). 4) Each chromatin has its own territory in the nucleus, in which locus from the same chromatin have higher probability to localize together and form exclusive subregions (Stam et al., 2019). In the normal cell nucleus, euchromatins, which are gene-rich and transcriptionally active segments, cluster together in the interior, whereas heterochromatins, which are gene-poor and silenced, cluster together in the nuclear envelope. Visualization in real time and simulation in silicon provides a comprehensive understanding of the phase separation and chromosome territory (Liu et al., 2020; Oliveira Junior et al., 2020; Su et al., 2020). Overall, chromatin is organized in a highly hierarchical architecture. Each of these four layers is also highly dynamic, which provide potential to regulate important biological processes, in particular the gene expression.

The human genome is the blueprint of life consisting of more than 20,000 genes and millions of regulatory candidate elements (Dunham et al., 2012). Despite intensive efforts, it is far from complete to understand how these elements function and interact with each other in the spatial and temporal dimensions to regulate gene expression, as cells with identical DNA sequence can function differently. The three-dimensional structure and dynamics of chromatin play critical roles in bringing into physical proximity the regulatory elements with target genes across hundreds of kilobases or even megabase distance. The abnormal chromatin organization leads to the occurrence of diseases, in particular cancer (Goes et al., 2011; Meaburn et al., 2016). 3C-based methods such as Hi-C (Dekker et al., 2002; Lieberman-aiden et al., 2009) and FISH (Fraser et al., 2015; Fudenberg and Imakaev, 2017) are two mainstream experimental techniques to probe the organization and dynamics of chromatin. However, how to integrate and interpret the Hi-C and the FISH measurements remains challenging, and in some cases, they can even lead to contradictory results (Fudenberg and Imakaev, 2017).

Complementary to experiments, computer simulations provide unprecedented resolution to investigate the folding of chromatin. Polymer model theory can be used to study the organization and dynamics of chromatin fibers at genome scale. Coarse-grained (CG) models of nucleosome can be used to study the interaction and dynamics of nucleosomes array which is the local subregion of the chromatin fiber. All-atom molecular dynamics (MD) simulations of nucleosome can provide further detailed information at the atomistic level. Combining these models for multiscale simulations would be useful to enhance our understanding of chromatin folding processes. In the polymer model (Philip et al., 1993; Münkel and Langowski, 1998), chromatin is represented as polymers consisting of different monomers connected by harmonic bonds, and different persistence lengths are chosen according to the compaction ratio, for example, how many base pairs are coarse-grained into one monomer. The models also account for particular interactions related to the biological activities of chromatin, for example, loop extrusion and compartmental segregation. Recently, two sophisticated computational models with slightly different potential energy function formulas have been developed (Di Pierro et al., 2016; Fudenberg et al., 2016), and shed light on the mechanisms underlying the folding and organization of chromatin (Gibcus et al., 2018; Mirny et al., 2019; Stam et al., 2019; Banigan et al., 2020).

The quality of polymer model simulations depends critically on the accuracy of their potential energy functions, which are a summation of different pairwise interaction terms. Previous researches (Sanborn et al., 2015; Fudenberg et al., 2016; Goloborodko et al., 2016a; Goloborodko et al., 2016b) typically used grid search strategy to optimize the parameters in potential energy functions, which means trial-and-error until simulated properties match experiments. If there are four parameters in one energy function and each parameter have 10 grids, 10,000 sets of parameter combination are tried from which one will be selected, the one most consistent with the Hi-C contact map. In addition to grid search strategy, the maximum entropy principle (Zhang and Wolynes, 2015; Di Pierro et al., 2016) was also applied to derive the potential functions using the experimental contact map information as inputs. A prior knowledge is needed to derive models from the maximum entropy principle, where it is used by grid search strategies as the criterion to select the best parameter combination. An alternative way to construct coarse-grained models is using the “bottom-up” or “*ab initio*” approach. One can derive coarse-grained physical potentials from more detailed simulations, for example, explicit-solvent all-atom MD simulations (Noid et al., 2008; Li et al., 2016). Here, we present a first step toward constructing a multiscale polymer model for chromatin based on atomistic simulations of two nucleosomes.

Nucleosomes are fundamental units for chromatin folding and the main carrier of epigenetic marks, so we coarse-grain one nucleosome as one bead in the coarse-grained model. Since the high-resolution X-ray structure of the nucleosome was determined in 1997 (Luger et al., 1997), there have been many studies on the properties of nucleosomes (Portela and Esteller, 2010; Biswas et al., 2013; Feng and Li, 2017; Zhou et al., 2019). Recently, Funke et al. (2016) employed force spectrometer and single-particle electron microscopy to measure the forces and interaction profiles between nucleosomes by placing two nucleosomes close to each other in a variety of defined relative orientations and recording the frequency of their distances. Multiscale simulations revealed that increased secondary structure resulting from acetylation of H4 tail has an important effect on the rigidification and also impaired the interactions between stacked nucleosomes (Collepardo-Guevara et al., 2015). The effect of H4 tail on stabilizing the stacked nucleosome is also validated by a recent first atomistic simulation of stacked nucleosomes using conventional and steered MD simulations (Saurabh et al., 2016). Ishida and Kono (2017) explored the energy landscape of two stacked nucleosomes using umbrella sampling with nucleosomes restrained at a few distinctive orientations. Moller et al. (2019) evaluated the anisotropic energy landscape of stacked nucleosomes across a variety of parameters in configurational and environmental space using residue coarse-grained simulations. Lequieu et al. (2019) constructed a coarse-grained multiscale model of chromatin by mapping the energy landscape of stacked nucleosomes to a reduced coarse-grained topology. Interestingly, Spakowitz and co-workers investigated the effects of epigenetic modifications, especially methylation of lysine-9 of histone H3, on the organization and dynamics of chromatin using polymer model (MacPherson et al., 2018; Sandholtz et al., 2020).

Nucleosome positioning, referring to the location of the nucleosome dyads in linear DNA, regulates the accessibility of DNA to DNA-bound proteins (Portela and Esteller, 2010). Adjacent nucleosomes in sequence are connected by linkers, whose lengths are about tens of base pairs in eukaryotes. The average radius of folded protein is about a few nanometers (Milo et al., 2009), whereas the compartment structures are across megabases. So the process of compartmentalization is self-organized by the interactions between nucleosomes, rather than mediated by proteins. Modifications of nucleosome, such as DNA methylation and histone modification, change the properties of nucleosomes and thus alter their interaction landscapes. Theoretical modeling of the interactions between two nucleosomes, in particular its dependence on the distance and relative orientation of nucleosomes, are crucial to our understanding of chromatin folding.

In this work, we present all-atom MD simulations of two nucleosomes interacting with each other in physiologically relevant explicit-solvent environment and analyze their interaction landscapes in the context of the 30-nm chromatin model. We consider two simulation systems, one containing two linked nucleosomes, whereas the other one containing two unlinked nucleosomes proximal in space. Atomistic simulation results of these two systems could be used to determine the function forms and parameters in the model, such as the diameter of the beads, the strength of the harmonic bonds connecting beads, and the strength of the weak attraction between beads in different chromatin states. The manuscript is organized as follows: Details of MD simulations and trajectory analysis will be provided in the Methods section. In the Results section, unbiased MD simulation results will first be presented, followed by the potential of mean force (PMF) and coarse-graining calculations. The manuscript ends with a short discussion and conclusion.

## Methods

### System Setup

**FIGURE 1:**
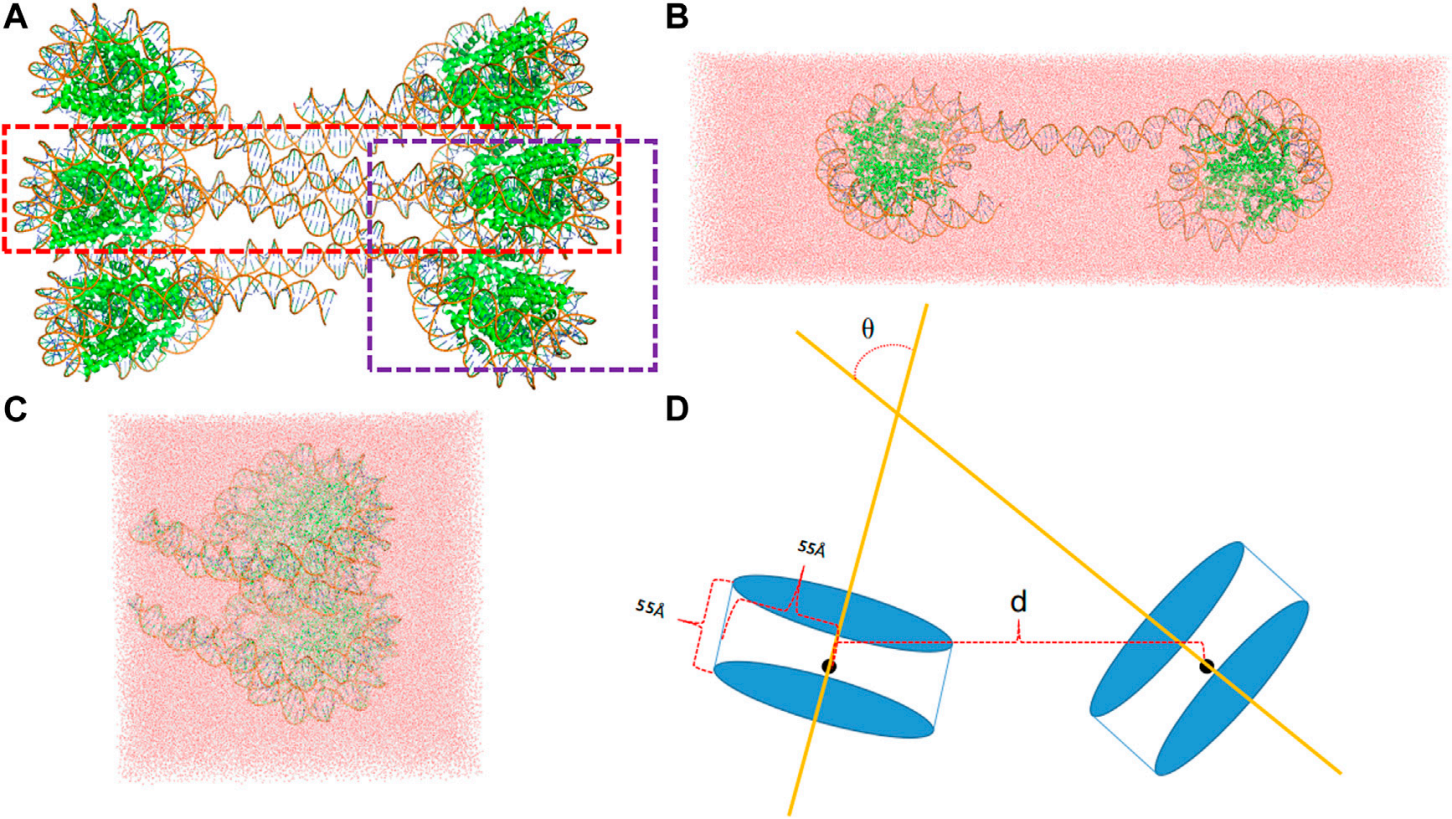
**(A)** The template structure (pdb id: 6hkt) for the setup of simulation system includes six nucleosomes with a flat two-start helix with uniform nucleosomal stacking interfaces and an uncondensed nucleosome packing density. The LN system is marked by a red rectangle, whereas the ULN by a purple rectangle. **(B)** A snapshot of the LN system after 10 ns MD simulation. **(C)** A snapshot of the ULN system after 10 ns MD simulation **(D)** The distance, *d*, defined using the centers of nucleosomes and the angle, *θ*, defined using the superhelical axes of nucleosomes.

The interaction between two nucleosomes can be naturally divided into two types, one type representing the interactions between nucleosomes that are connected by the linker DNA and the other type representing the interactions between two spatially adjacent nucleosomes that are stacked together with no connecting linker. Accordingly, two simulation systems were set up as shown in Figure 1. The initial structures were taken from that of classical 30 nm fiber (pdb id: 6hkt) (Garcia-Saez et al., 2018). Although its resolution is relatively low (9.7 Å), it includes the information about the nucleosomal stacking and packing patterns which are important for building the simulated systems to model the interactions between nucleosomes. We note that the histone tails that are missing in the crystal structure are not modeled in the simulation systems.

For the linked-nucleosomes (LN) system, two nucleosomes and their corresponding linker DNA base pairs were extracted from the experimental structure and solvated in an explicit TIP3P (Jorgensen et al., 1983) water box with dimensions of 463 Å × 142 Å × 110 Å (Figure 1B). CHARMM (Brooks et al., 1983; Brooks et al., 2009) was used to build the missing hydrogen atom coordinates and patch protein and nucleic acid terminals. 150 mM KCl was added with additional cations to neutralize the system. In total, the LN system contains 676,742 atoms which are composed of 16 protein chains, two DNA chains, 209,471 water molecules, 1,140 K^+^ ions, and 594 Cl^−^ ions.

The unlinked-nucleosome (ULN) system contains two stacked nucleosomes plus 15 flanking base pairs extracted from the experimental 30 nm fiber structure. A similar procedure was used to solvate the system in a cubic water box with dimensions of 182 Å × 182 Å × 182 Å (Figure 1C). In total, the ULN system has 558,630 atoms composed of 16 protein chains, four DNA chains, 170,064 water molecules, 1,039 K^+^ ions, and 485 Cl^−^ ions. Proteins and DNAs were modeled by the CHARMM36m protein force field (Huang et al., 2017) and the CHARMM36 nucleic acid force field (Hart et al., 2012), respectively.

### Molecular Dynamics Simulations

MD simulations were performed using OpenMM (Eastman and Pande, 2010; Eastman et al., 2017) with the isothermal–isobaric (NPT) ensemble. Periodic boundary condition (PBC) was applied and particle mesh Ewald (PME) (Essmann et al., 1995) was used to compute all the nonbonded interactions with a real space cutoff at 9 Å. We noted that both electrostatics and van der Waals interactions are fully accounted for with no truncations, as the latter were treated by the recently developed LJ-PME method (Wennberg et al., 2015). All hydrogen-containing bonds were constrained by the SETTLE algorithm (Miyamoto and Kollman, 1992) and the Verlet integrator was used with a time step of 2 fs The temperature was maintained at 303.15 K using the Andersen thermostat (Andersen, 1980) with a damping coefficient of 1 ps^−1^. A Monte Carlo barostat (Aqvist et al., 2004) was used to maintain the pressure at 1 atm by attempting to change the box dimension every 25 steps. Both LN and ULN systems were simulated to 1 µs, and frames were saved every 5,000 steps (10 ps).

### Umbrella Sampling and Potential of Mean Force Calculations

To investigate the free energy landscape between nucleosomes, advanced simulation techniques need to be employed. Here, we perform umbrella sampling simulations using the distance between the two nucleosomes as the reaction coordinate.

$$H_{\mathrm{umb}}=H_{0}+\frac{1}{2}k{\left(d-d_{0}\right)}^{2},$$ (1)

where the system Hamiltonian $H_{0}$ is biased by a harmonic potential that restrains the select reaction coordinate *d* at a certain value $d_{0}$. Biased MD simulations employing Eq. 1 with different $d_{0}$ values (windows) can be carried out to enhance the sampling of events hindered by free energy barriers. The initial configuration for each window was extracted from the unbiased MD simulations with *d*, most close to the targeted $d_{0}$ value. The NVT ensemble was used for umbrella sampling. The system was equilibrated for 1 ns with no biased potential added and then subject to 30 ns umbrella sampling simulations.

Umbrella sampling simulations were carried out using OpenMM with a plugin for PLUMED (Tribello et al., 2014). The center of a nucleosome was defined as the center of mass of the phosphorus atoms of DNA wrapped on the nucleosome histone cores (LN system) or the center of mass of protein C_*α*_ atoms of the histone cores (ULN system). The reaction coordinate *d* was then defined as the distance between the centers of nucleosomes. For the LN system, 127 windows were used in total, $d_{0}$ ranging from 175 to 250 Å with an interval of 1 Å and additional windows ranging from 187.5 to 237.5 Å with an interval of 1 Å. For the ULN system, 68 windows were used in total, $d_{0}$ ranging from 60 to 91 Å with an interval of 0.5 Å and additional five windows ranging from 55 to 59 Å with an interval of 1 Å. For the LN system, 5 kJ/mol/Å^2^ was selected as the value of *k*, whereas 10 kJ/mol/Å^2^ was used in alternative windows for the ULN system. Good phase space overlap between windows was achieved (Supplementary Figures S1, S2). The potential of mean force profiles were calculated using the weighted histogram analysis method (WHAM) (Grossfield, 2020–9).

## Results

### Unbiased Molecular Dynamics Simulations

**FIGURE 2:**
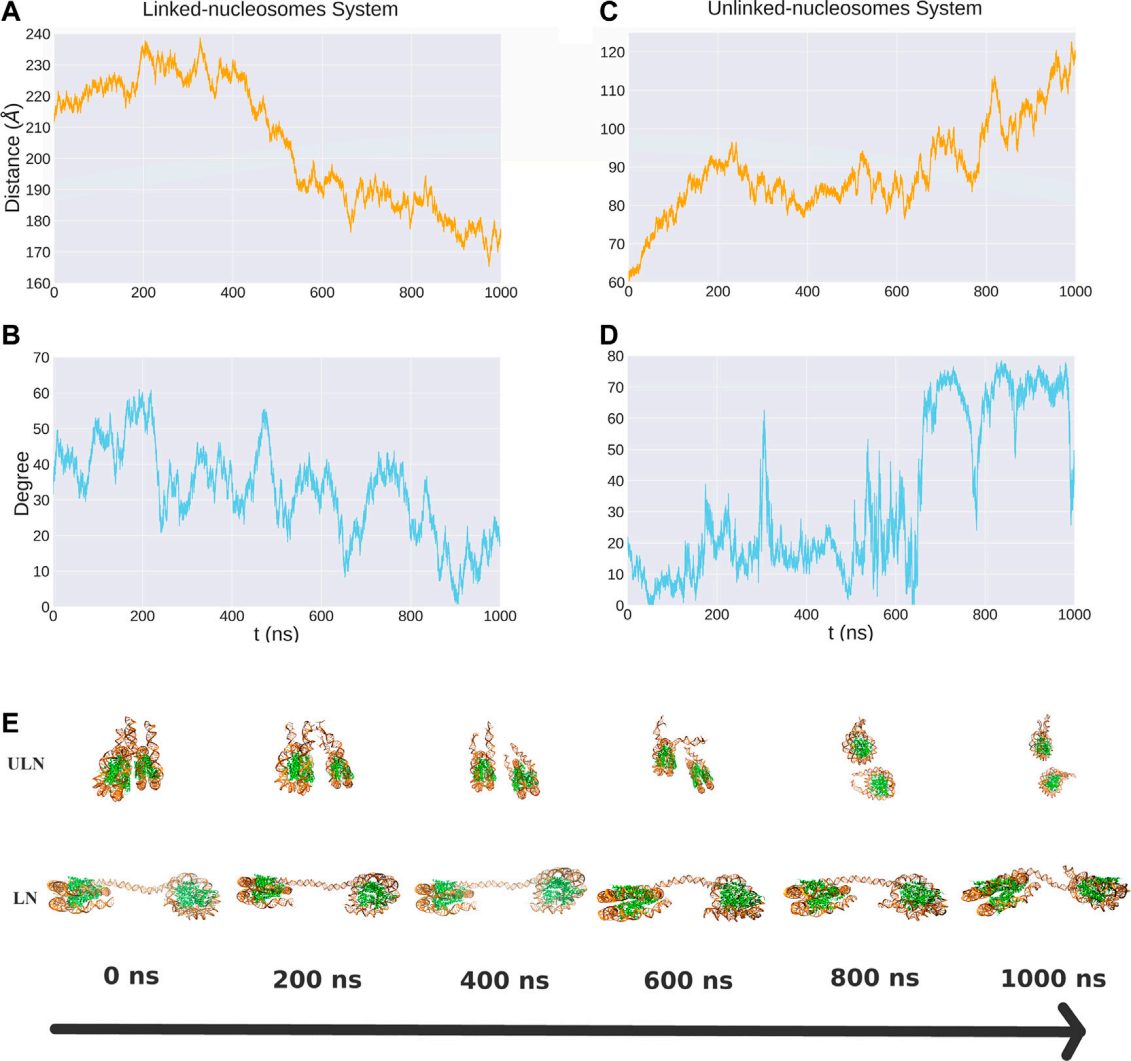
The distance and relative orientation between two nucleosomes in the LN **(A, B)** and ULN **(C, D)** systems from 1 *μ*s unbiased MD simulations. Also shown are snapshots corresponding to different time points **(E)**.

Unbiased MD simulations were carried out for 1,000 ns for both LN and ULN systems. For individual nucleosome, the binding between DNA and histone core was very stable as indicated by their root mean square deviations (RMSDs) being around 4 Å with respect to the initial structures (Supplementary Figure S3). The relative motion between nucleosomes, in contrast, was highly dynamic with respect to both their distance and orientation over the microsecond timescale (Figure 2). For nucleosomes connected by the linker, their distance *d* varied between 165 and 239 Å. The distance increased to 238 Å in the first 200 ns of the simulations and gradually decreased to 170 Å after 400 ns.

For unlinked nucleosomes, *d* increased almost linearly from the starting value of 60 to about 90 Å at the first 200 ns, and then fluctuated between 75 to 100 Å in the following 600 ns, and rose to 120 Å after 800 ns. This suggests that the equilibrium distance of two free nucleosomes in aqueous environment is much larger than the 62 Å in the crystal structure of 30 nm fiber, which are impacted by the crystal packing and the stacking of nucleosomes. Based on our simulations, the relative velocity between two nucleosomes can be estimated to be about 0.1 Å/ns or 0.01 m/s.

We also analyzed the relative orientation between the two nucleosomes, characterizing it using the angle between the superhelical axes of nucleosomes (Figure 1D). There were large variations of angle in 1,000 ns MD simulations for both LN and ULN systems. The length of the linker DNA in the linked-nucleosomes system is 40 base pairs, smaller than the persistence length of double helix DNA (about 150 base pairs). A weak correlation between orientation and distance *d* was observed in both the LN and ULN systems. In the LN system, the relative orientation angle has a tendency to decrease when *d* decreases. In the ULN system, the angle has a tendency to increase with the distance. The frequency of angle variation is significantly higher than that of distance. During 1,000 ns MD simulation, distance went down in the LN or up in the ULN system, whereas the angle vibrated up and down regularly.

### Interaction Free Energy Landscapes

**FIGURE 3:**
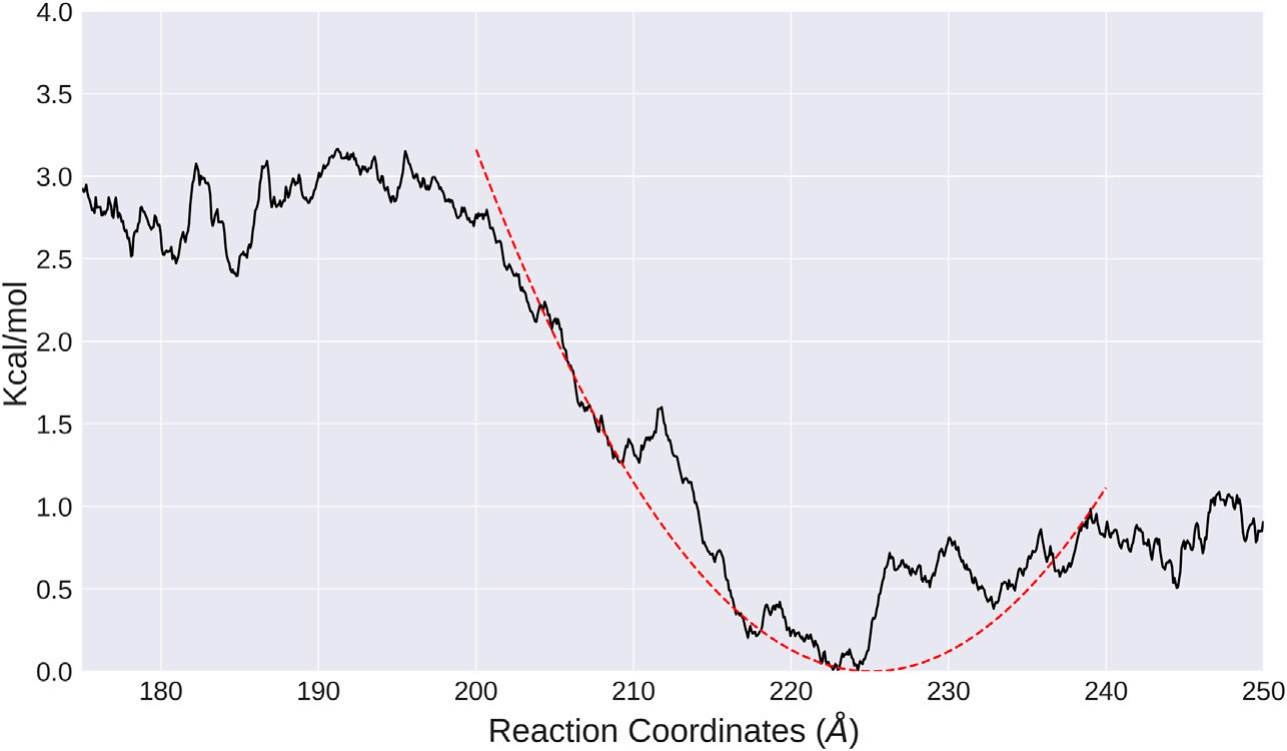
PMF calculated from umbrella sampling in the LN system along the distance between two nucleosomes (black solid line). The fitted harmonic potential function is shown as red dotted line.

To study the effective interactions between nucleosomes in both systems, umbrella sampling simulations were performed to compute the PMFs as a function of distance between the nucleosomes. In general, the free energy profiles are shallow and flat, consistent with the hypothesis that nucleosomes are highly dynamic. The convergence of PMF is relatively good for both LN and ULN systems as indicated by the statistical uncertainties from the Monte Carlo bootstrapping calculations with WHAM (Grossfield, 2020–9) being low (Supplementary Figures S8–S11). For the LN system, the global minimum is found at d=224 Å. Fitting the PMF around this minimum into a harmonic potential led to an equilibrium distance of 225 Å and a vibrational frequency of $2.41\;\times \;10^{9}\;s^{-1}$. As shown in Figure 3, the interaction free energy increases harmonically when the two nucleosomes are compressed from the global minima and then becomes flat and rugged below 200 Å, whereas the interaction free energy increases slightly and then becomes flat and rugged. This suggests that the linker DNA is more like a rubber string other than a spring. We also analyzed the relative orientation of nucleosomes in each window. The orientation angle along the time series in each window was stable and vibrated regularly within a small interval (Supplementary Figure. S4), whereas the average values were strongly dependent on the reaction coordinate *d* (Supplementary Figure. S5). The umbrella sampling results are consistent with the observations from unbiased MD simulations (Figure 2A), which provide more quantitative information on the interaction landscape of linked nucleosomes.

**FIGURE 4:**
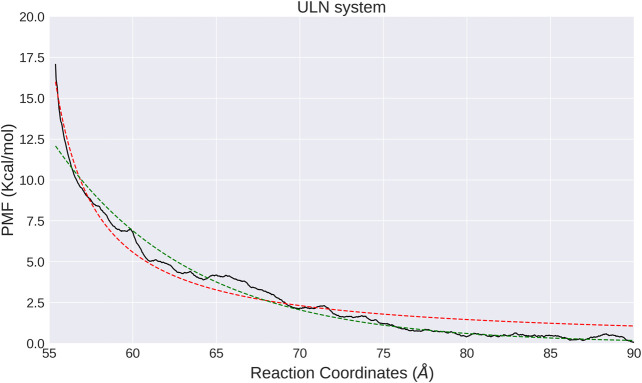
PMF calculated from umbrella sampling in the ULN system along the distance between two nucleosomes (black solid lines). The fitted exponential and shifted Coulomb potential functions are shown as red dotted line and green solid line, respectively.

The interaction between two unlinked nucleosomes in solution has a completely different free energy profile, featured by a strong repulsion wall at smaller distances, and a flat curve for larger *d* (Figure 4). It indicates that the interactions between ULN nucleosomes are repulsive. As histone tails are not included in our ULN simulation system, the repulsive interaction is consistent with previous simulations (Collepardo-Guevara et al., 2015; Saurabh et al., 2016; Ishida and Kono, 2017; Moller et al., 2019) and experiments (Dorigo et al., 2003; Gordon et al., 2005), which showed histone tails are crucial for holding stacked nucleosomes together. We note that the nucleosome has a disc-like shape with a height of 55 Å, so the sharp free energy rise below *d* = 60 Å is probably not caused by steric clash but instead by the unfavorable electrostatic interactions as each individual nucleosome is highly negatively charged. The asymptotic free energy was not fully resolved in our calculations, due to the limited size of the simulation box (182 Å × 182 Å × 182 Å). When the two nucleosomes are pulled away with *d* larger than 90 Å, interactions with their PBC images become possible such that the WHAM analysis is no longer valid.

Similar to the LN system, we also analyzed the orientation of nucleosomes in each window. The relative orientation angle along the time series in each window is stable and vibrates regularly at a small interval (Supplementary Figure. S6) and correlates with the distance *d* (Supplementary Figure. S7). In the ULN system, orientation angle has a tendency to increase with *d* and has larger fluctuation at larger *d*, consistent with unbiased MD simulation in which angle increases with distance (Figures 2C,D). The computational PMF for the ULN system can be directly compared with a recent experimental measurement of the association of nucleosomes (Funke et al., 2016). By integrating two nucleosomes into a carefully designed and calibrated DNA origami-based force spectrometer, Funke et al derived the Boltzmann-weighted distance-dependent energy landscape for two nucleosomes interacting with each other. They observed three major features: a strong repulsion at distances smaller than 60 Å; a minimum located somewhere between 60 and 70 Å; and vanishing interactions at distances greater than 130 Å. The computational PMF shown in Figure 4 agrees well with strong repulsion at small distances and vanishing interactions at distal distance. No global minimum is found in our PMF due to the absence of histone tails in the ULN system, suggesting the importance of histone tails from an indirect perspective.

### Constructing Coarse-Grained Potentials

With umbrella sampling, we are able to dissect the interactions between nucleosomes with atomistic details. On the other hand, the human chromatin contains about 28 million nucleosomes, so it is not feasible to use atomistic simulations to study chromatin folding in the foreseeable future. The free energy profiles we obtained can serve as the starting point to construct coarse-grained potentials to study the conformational dynamics of chromatin elements such as 30 nm fibers. Current polymer theory models for chromatin (Di Pierro et al., 2016; Fudenberg et al., 2016) are typically composed of five terms:

$$H_{\text{total}}=\sum^{\text{​}}H_{\text{bond}}+\sum^{\text{​}}H_{\text{stiffness}}+\sum^{\text{​}}H_{\text{loop}}+\sum^{\text{​}}H_{\text{compartment}}+\sum^{\text{​}}H_{\text{permeability}},$$ (2)

where the $H_{\text{bond}}$ term models the interaction between adjacently connected monomers, $H_{\text{stiffness}}$ describes the rigidity of chromatin fibers, $H_{\text{loop}}$ term represents the biological process of loop extrusion, $H_{\text{compartment}}$ term models the compartmentalization, and $H_{\text{permeability}}$ models the biological function of topoisomerase II that makes DNA free of knots. In general, the $H_{\text{bond}}$ and $H_{\text{stiffness}}$ are common items that are intrinsic in the conformation of chromatin and conserved over the cells of different sources and species. The mechanism behind the $H_{\text{loop}}$ item is complicated because how the involved SMC complexes establish the loop structure at the molecular level remains unknown, especially a cell may have different $H_{\text{loop}}$ at different states. $H_{\text{compartment}}$ is highly correlated with the interactions between unlinked nucleosomes and epigenetic modifications on histone cores and can describe the phase separation responsible for the compartmentalization. Here, we derived $H_{\text{bond}}$ and $H_{\text{compartment}}$ items using all-atom MD simulations.

If we construct a CG model in which each nucleosome is coarse-grained into one monomer, the $H_{bond}$ term can then be directly inferred from the atomistic simulation results of the LN system. As shown in Figure 3, the PMF can be fitted with a harmonic potential:

$$V\left(d\right)=\frac{1}{2}k{\left(d-d_{0}\right)}^{2},$$ (3)

where *k* equals 0.01 kcal/mol/Å^2^ and $d_{0}=225.0\;\text{Å}$.

The free energy profile of the ULN system shows that the interactions between a pair of nucleosomes in distant distance are very weak (Figure 4). This suggests that the nucleosomes might not self-aggregate if there are no additional restraints such as histone tail effects, epigenetic modifications or binding of proteins such as cohesins or condensins. $H_{\text{compartment}}$ in Eq. 2 models compartmentalization, which means that nucleosomes in different compartments (A and B) have different interaction strength (Di Pierro et al., 2016). In general, A compartments match euchromatins and B compartments correspond to heterochromatins. The interaction landscape of two free, unmodified nucleosomes from explicit-solvent all-atom MD simulations would be useful to construct some of the $H_{\text{compartment}}$ terms. If we use an exponential potential to fit the PMF

$$V\left(d\right)=e^{-\alpha \cdot d-\beta }$$ (4)

we could determine the parameters to be $\alpha =0.1213\;{\text{Å}}^{-1}$ and β=9.2086 (red line in Figure 4).

Another suitable potential energy function form to fit the PMF would be a shifted Coulomb potential.

$$V\left(d\right)=\frac{A}{d-B}.$$ (5)

However, fitting the PMF with such a shifted Coulomb potential leads to A=39.53 kcal/mol⋅Å and B = 52.82 Å (green line in Figure 4).

## Discussion and Conclusion

A spatially and temporally resolved understanding of chromatin organization is currently one of the central topics in molecular and cell biology. Here, we used classical MD simulations and enhanced sampling methods to study the basic element of chromatin, nucleosome. In particular, we studied the conformational dynamics and the free energy landscapes between two nucleosomes in aqueous environment. Not only the widely investigated stacked nucleosomes but also the linked nucleosomes are involved in our study. With umbrella sampling calculations, we obtained detailed free energy profiles for nucleosome pairs either free or connected by a linker DNA. Our simulation results compare favorably with a variety of experimental findings and MD simulations, in particular the PMF of the ULN system correlates well with a recent single-molecule force spectrometer measurement.

Considering that the configurations of nucleosomes are highly dynamic, the relative orientations of the stacked nucleosomes are often restrained to achieve quick convergence in previous simulation studies. In this work, the ULN (stacked) system was simulated freely without additional restrains at the cost of longer simulations, which makes the dynamics more natural and nucleosomes free to explore possible relative orientations. Both unbiased and biased simulations of unlinked nucleosomes show that electrostatic repulsion dominates distance distribution in the absence of histone tails. In addition to the ULN system, we also studied the dynamics and free energy profiles of linked nucleosomes. Results of LN and ULN systems could provide new insights into the organization and dynamics of larger chromatin elements such as 30 nm fibers.

Umbrella sampling is a useful technique to investigate the effective interaction between biological macromolecules. Recently, Lai et al (2020) studied the free energy profile between two identical DNA double helices using extensive umbrella sampling. In this study, we carried out 1 μs conventional MD simulations and found out that the relative motion between nucleosomes is very slow. We then performed accumulatively more than 3.8 μs umbrella sampling simulations on systems with more than half a million atoms to understand the slow- and large-scale dynamics between nucleosomes. This is only possible with recent advances in both hardware and software that utilize the graphics processing units (GPUs) for MD simulations. Simulation of the LN system (∼ 677,000 atoms) runs about 10 ns per day on a single Tesla V100 GPU card. One advantage of umbrella sampling is that the method is naively parallel so simulations of different windows can be carried out on different GPU cards at the same time.

We note that K^+^ ions were used as the cation in our simulation systems, whereas salt concentration and composition are more complicated in physiological environment and will impact the strength of nucleosome interactions. Mg^2+^ ions were known to play a crucial role in directly binding with and stabilizing nucleic acids. However, more accurate polarizable force fields might be needed to model Mg^2+^ ions (Huang et al., 2014; Lemkul and MacKerell, 2016; Walker et al., 2020). It would be interesting to investigate how the interaction landscapes between nucleosomes change with the different ion strength and composition. Another flaw of the current study concerns the limited simulation box size. For the ULN system, we are not able to obtain the asymptotic free energy over distance larger than 90 Å, as nucleosomes will interact through their images due to the PBC conditions. A larger and probably noncubic water box might be needed to overcome these limitations.

We carried out atomistic simulations of nucleosomes with the long-term goal to construct a coarse-grained potential for 30 nm chromatin structure to bridge the gap between all-atom model of nucleosomes and polymer model. This would constitute a “bottom-up” approach to determine the function forms for polymer models and to optimize their parameters. With the analytically fitted CG potentials presented in this work, one would already be able to simulate, for example, the classical 30 nm chromatin fibers to investigate the stability and dynamics of such nucleosome fiber arrays and then optimize the polymer model with this coarse-grained potential. For this purpose, we built a system consisting of 100 nucleosomes which had no open ends and was covalently bonded to itself through periodic boundary conditions. When we propagated the simulation system using derived potential functions (Eqs 3 , 4) for $10^{6}$ steps (1 µs), the system was unstable and quickly crashed. In contrast, the structure remained stable if a weak attractive interaction between stacked nucleosomes was added mimicking the effect exerted by histone tails (Supplementary Figure S12). Unstability of 30-nm chromatin fiber with our CG model is consistent with previous research studies showing that histone tails are critical to hold nucleosomes together (Dorigo et al., 2003; Gordon et al., 2005).

This highlights one of the current limitations of the preliminary CG model presented here. Conversion between different compacted states of 30 nm chromatin significantly depend on the histone tails, especially H4 tails, so simulations with histone tails need to be carried out to derive the corresponding free energy profiles. Epigenetic modifications often occur on the histone tails, changing the properties of nucleosomes such as charge, rigidification, and solvent exchange which make a big difference in the dynamics and organization of chromatin and gene expression. Considering the importance of histone tails for both nucleosome stacking interactions and higher level chromatin structures, we are currently performing similar simulations with full histone tails. However, this involves significantly more extensive MD simulations that are out of the scope of the current study, which represents a first step toward building up a CG model for chromatin with a bottom-up approach. The binding between the linker histone and linker DNA also plays important roles in chromatin compaction (Luque et al., 2014), especially the interactions between linked nucleosomes. Similar linked nucleosomes systems with linker histone should be studied in future work. On the other hand, the model is not applied to any practical biological problem, for example, loop extrusion and compartmentalization. In reality, the disk-like shape of nucleosomes and nonuniform distribution of their charges make interactions highly anisotropic, whereas interactions are assumed to be isotropic in our coarse-grained model.

As for the next step, we will perform similar umbrella sampling simulations to study how epigenetic modifications on nucleosomes change the interaction landscapes and derive the corresponding potential parameters. Modifications of interests include acetylation of lysines on histone H4, mono-, di-, and trimethylation of lysine 27 on histone H3 and monoubiquitination of lysine 119 on histone H2A. Parameterization for these epigenetic modifications would allow us to construct a transferable computational model for chromatin. We plan to construct a coarse-grained model at nucleosome resolution, aiming to make the model as simple as possible so that it can be used to simulate one whole chromosome even genome which consists of millions of nucleosomes. Four or more beads (monomers) will be needed to intimate the anisotropicity of one nucleosome and the highly negative charge will be included simultaneously in the future. Ultimately, the polymer model will be optimized using “bottom-up” strategy. Such a model is expected to be useful in understanding development-related and disease-related chromatin dynamics, for example, how the binding of Polycomb repressive complex 1 (PRC1) and 2 (PRC2) (Comet et al., 2016) induces the condensation of chromatin.

## Data Availability

The original contributions presented in the study are included in the article/Supplementary Material, further inquiries can be directed to the corresponding author.
