# Supplementary material for: Interactions Between Nucleosomes: From Atomistic Simulation to Polymer Model
Source: Front Mol Biosci. 2021 Apr 12;8:624679. doi: 10.3389/fmolb.2021.624679 (PMC8072053; doi:10.3389/fmolb.2021.624679)
Supplement: Supplementary file 1 [file datasheet1.pdf]

Supplementary Material for
Interactions between Nucleosomes: from Atomistic Simulation to Polymer Model

1 FIGURES

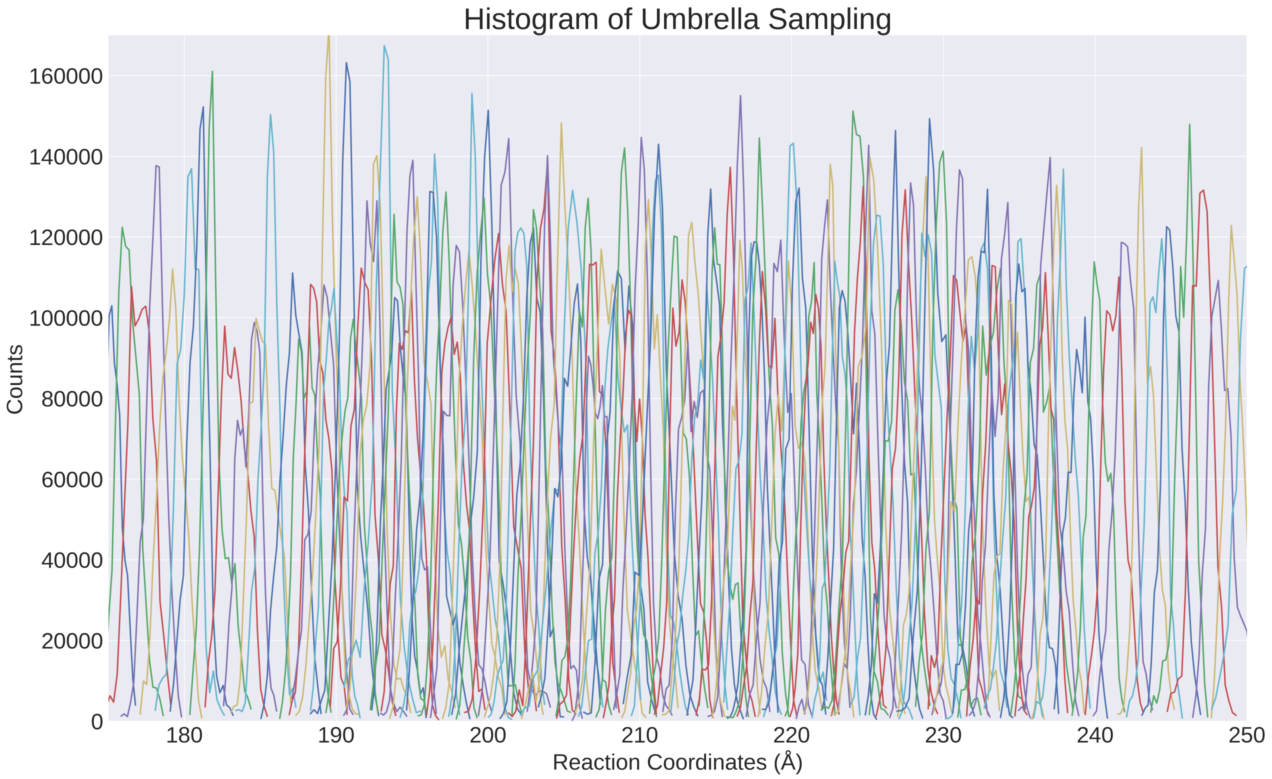

Figure S1. The distribution of reaction coordinates in umbrella sampling of LN system, which has 127 windows in total and the reaction coordinates, d, increase from 175 to 250 Å.

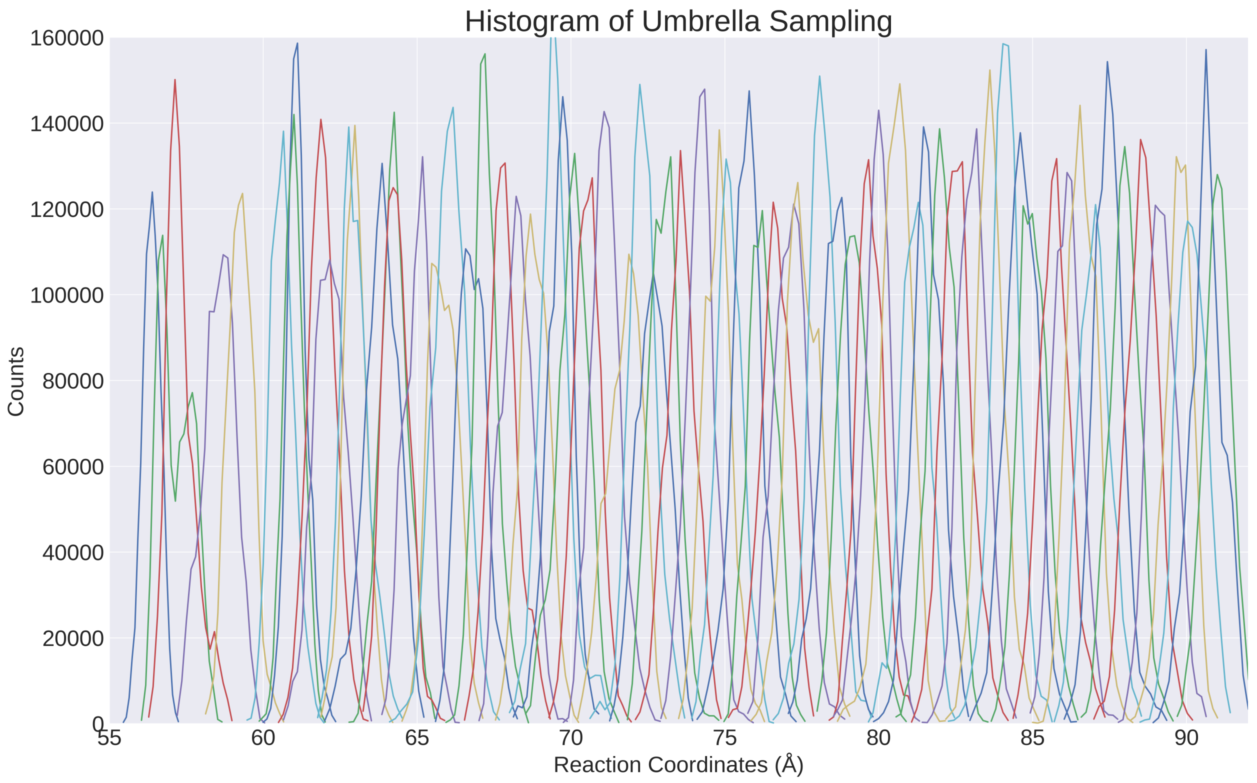

Figure S2. The distribution of reaction coordinates in umbrella sampling of ULN system, which has 68 windows in total and the reaction coordinates, d, increase from 55 to 91 Å.

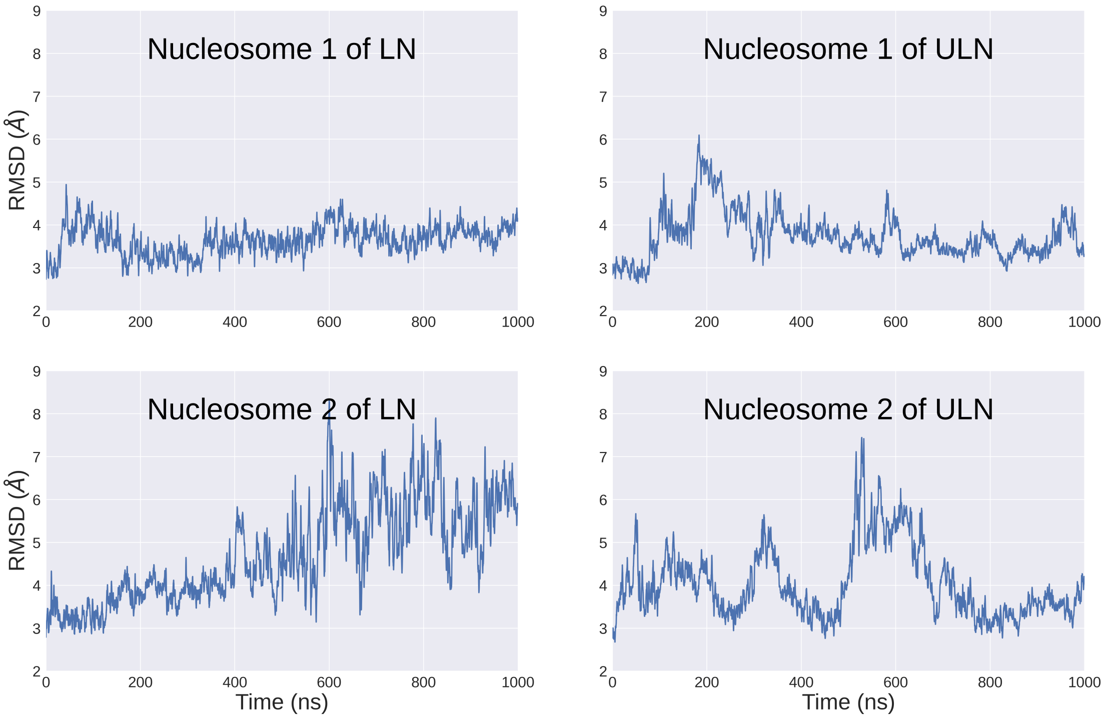

Figure S3. Both LN and UNL systems have two nucleosomes separately (see the Methods section and Fig. 1 in the main article). This figure shows the RMSDs of individual nucleosomes in the LN system (left panel) and the ULN system (right panel) along 1μs MD simulations.

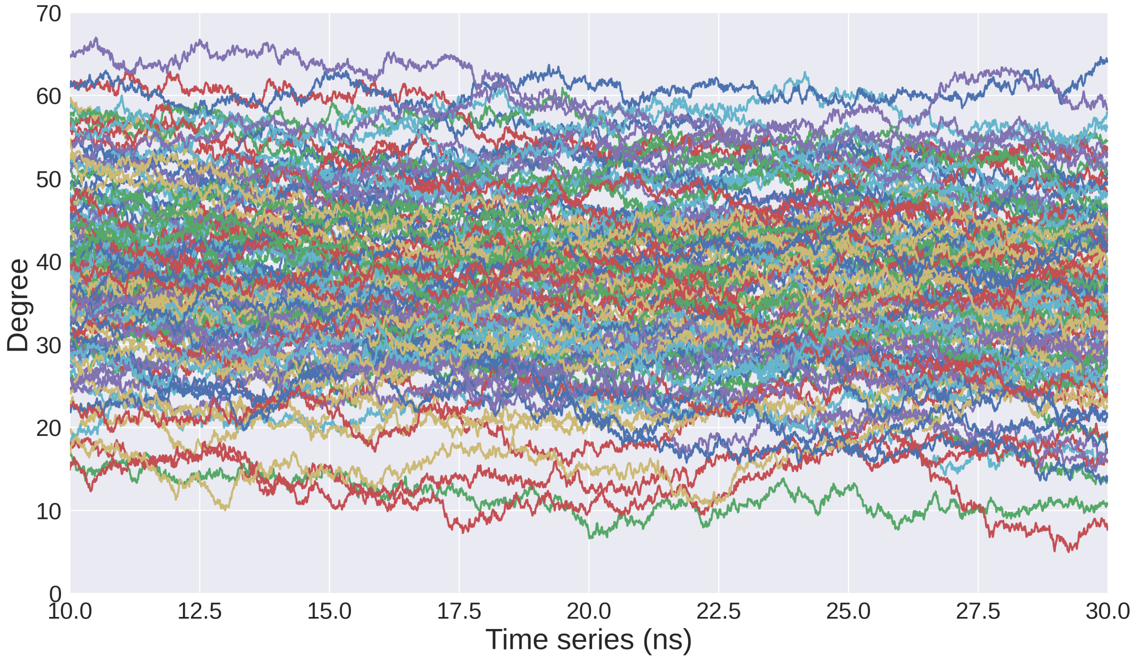

Figure S4. This figure shows degree along time series in each umbrella sampling window of LN system. In each run, the degree is stable and goes up and down regularly. In all runs, the degree ranges from 10 to 70.

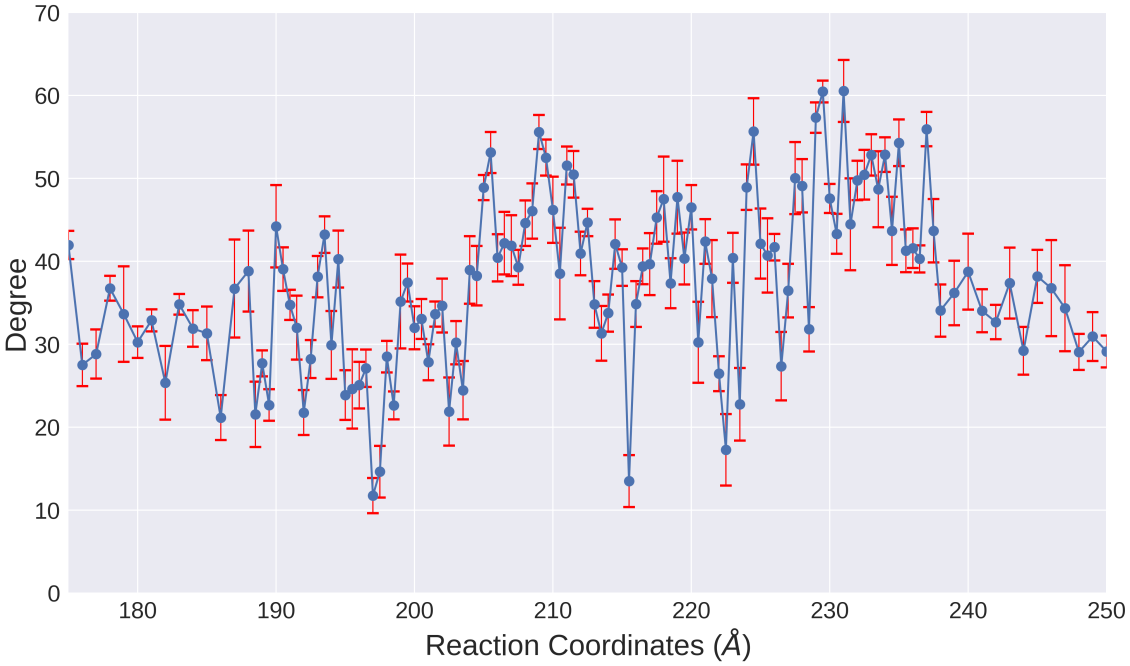

Figure S5. This figure shows the angle distribution along the distance and standard deviation of LN system. It seems that there is a positive correlation between angle and distance from 170 to 230 Å while a negative correlation above 230 Å.

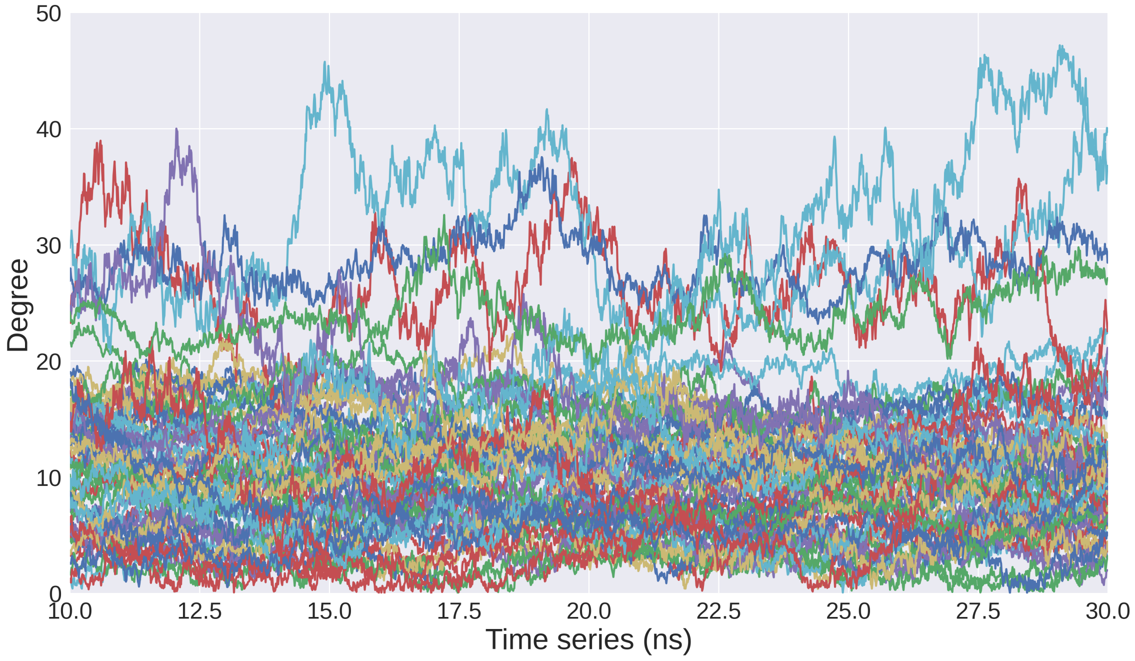

Figure S6. This figure shows degree along time series in each umbrella sampling window of ULN system. In each run, the degree is stable and goes up and down regularly. In all runs, the degree ranges from 0 to 50 and has larger amplitude of perturbation.

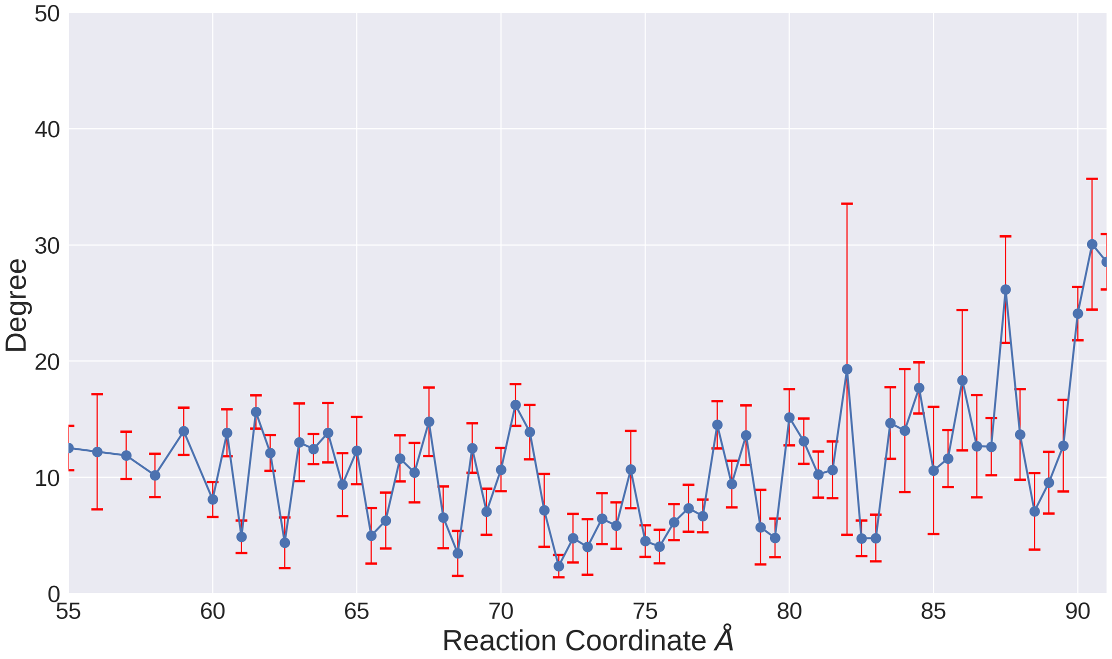

Figure S7. This figure shows the angle distribution along the distance and standard deviation of ULN system. It seems that angle has a tendency to increase with distance and has larger standard deviation at larger distance.

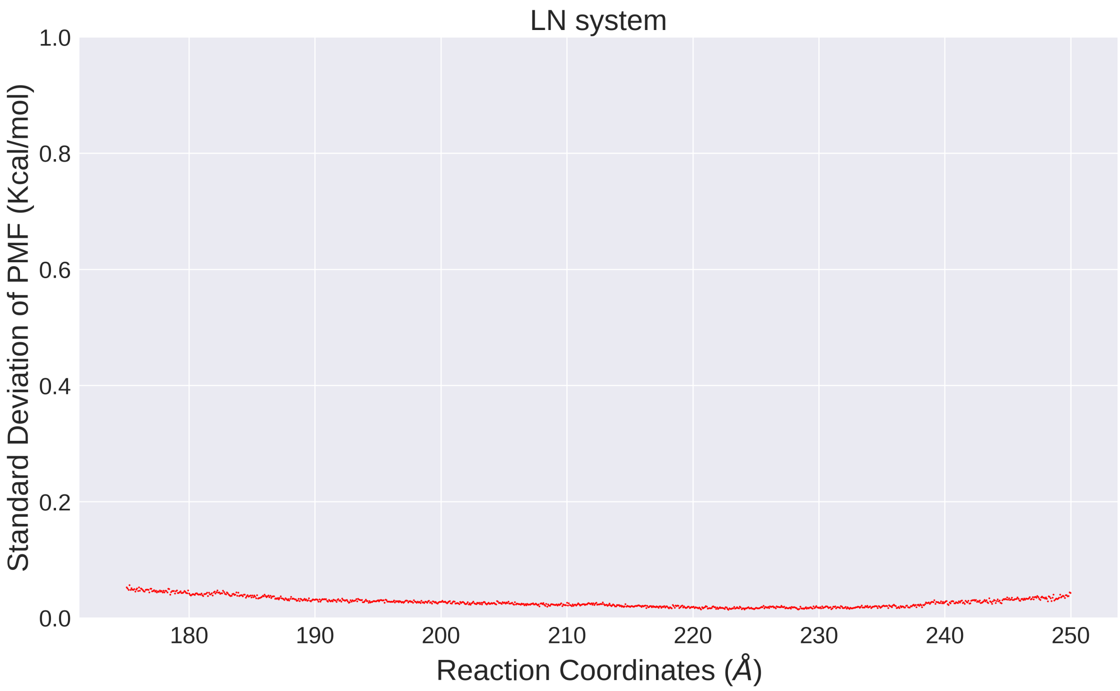

Figure S8. This figure shows the statistical uncertainty by Monte Carlo Bootstrapping in LN system using WHAM. It is very low.

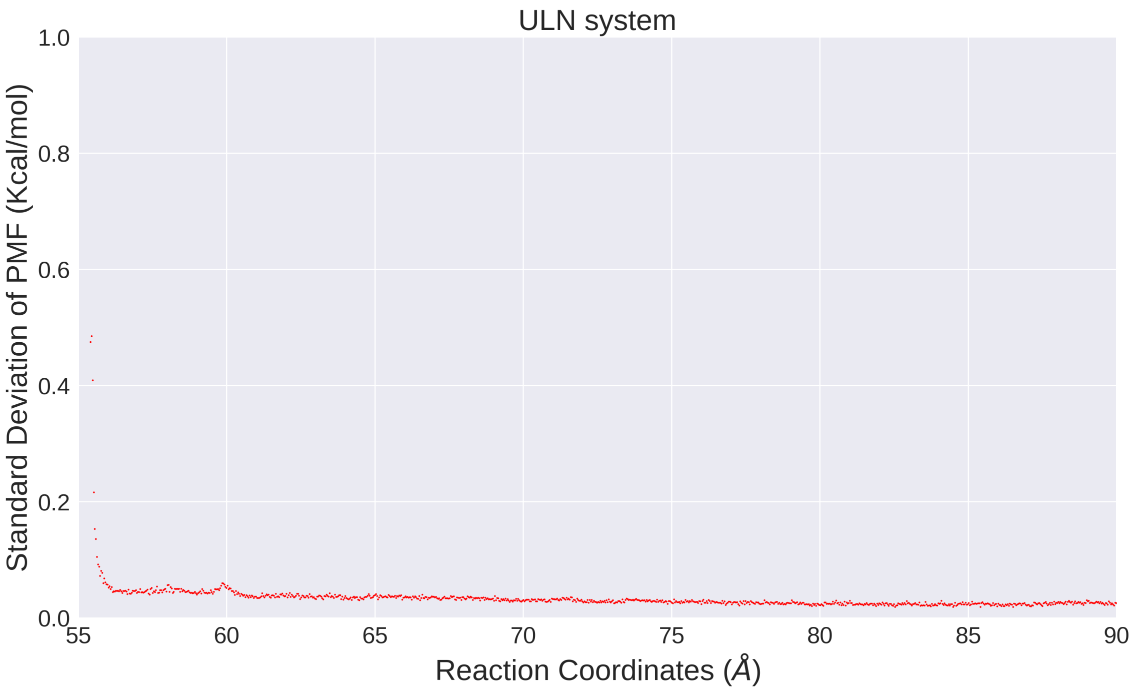

Figure S9. This figure shows the statistical uncertainty by Monte Carlo Bootstrapping in ULN system using WHAM. It is very low.

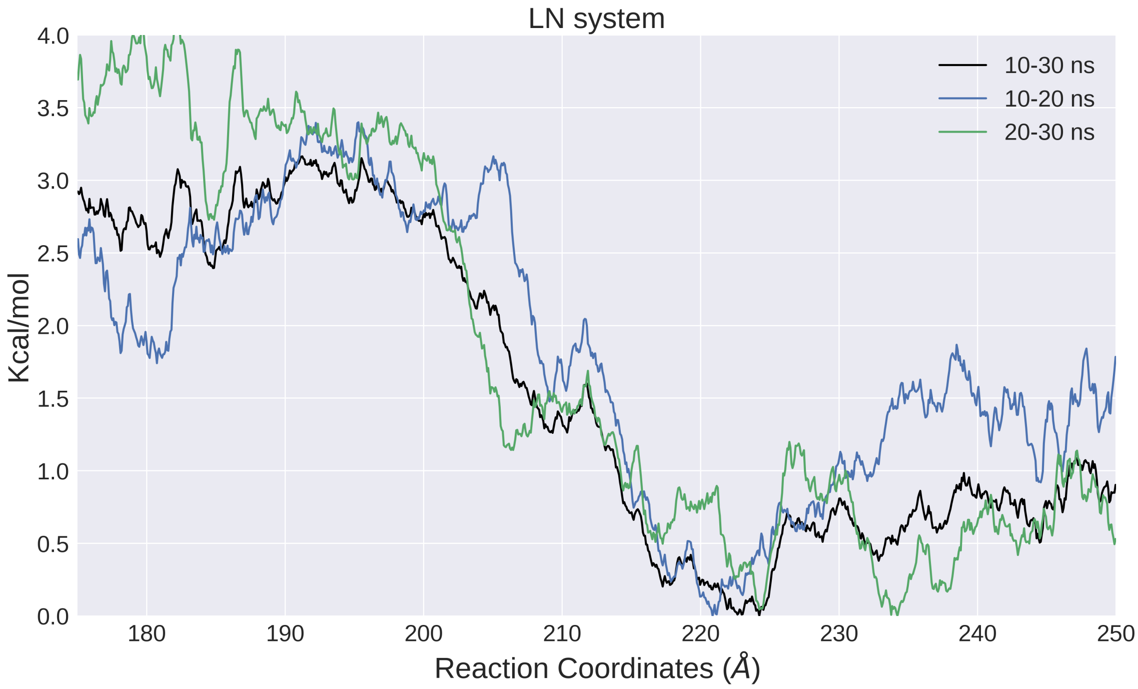

Figure S10. Each window was simulated for 30 ns. The first 10 ns trajectories was truncated and last 20 ns was used to calculate the PMF. To evaluate the convergence, we also calculate trajectories for 10-20 ns or 20-30 ns respectively. The variation trend is similar for 10-20 ns or 20-30 ns.

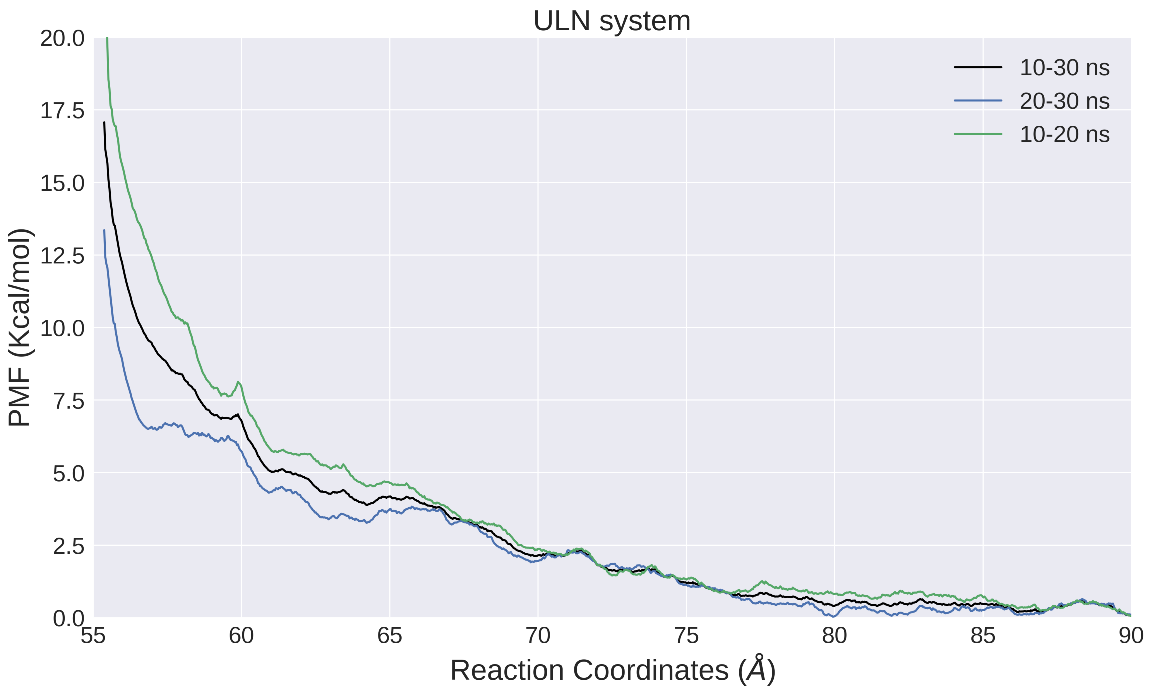

Figure S11. Each window was simulated for 30 ns. The first 10 ns trajectories was truncated and last 20 ns was used to calculate the PMF. To evaluate the convergence, we also calculate trajectories for 10-20 ns or 20-30 ns respectively. The PMF of ULN system has a good convergence.

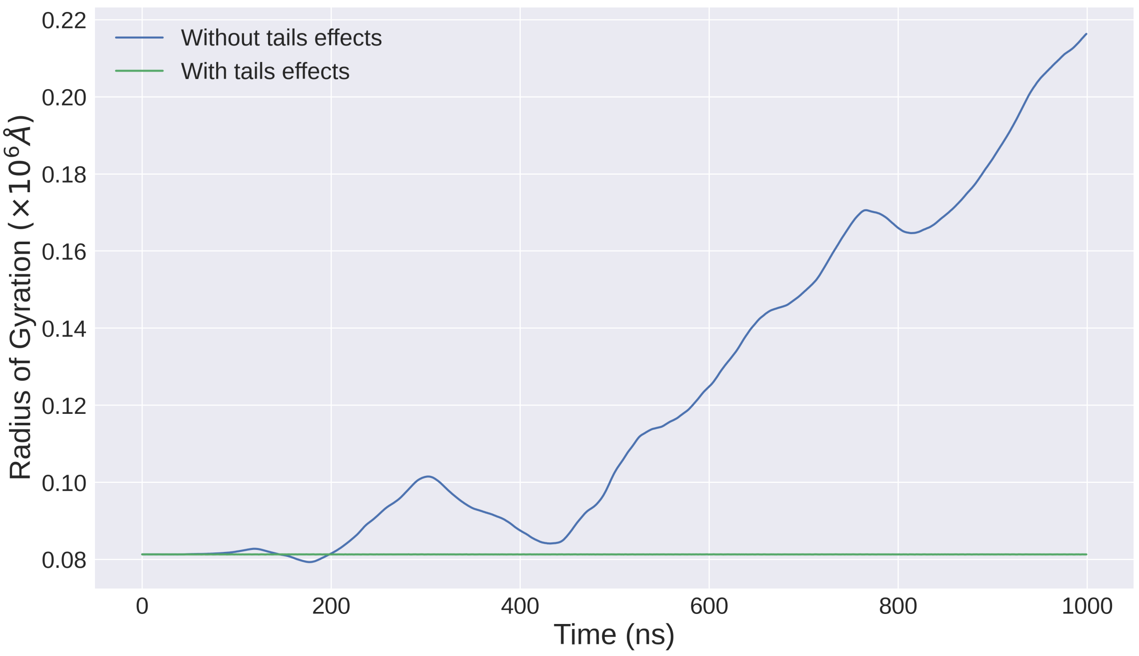

Figure S12. The time series of the radius of gyration for 30 nm fiber systems with (green) and without (blue) histone tail effects. The initial structure of 30 nm chromatin structure was built by mapping the monomers to the crystal structure (PDB code: 6HKT) and simulations were performed with a time step of 1 ps. It is not stable for the computational model present in this work, however it remains stable over 1μs time scale if harmonic bonds with $k = 0.05kJ/mol/\AA^2$ are added between stacked nucleosomes to represent the effect of histone tails.

Python script for running simulations with CG model in OpenMM:

```
#!/usr/bin/env python
# Chengwei Zhang(zhangchengwei@westlake.edu.cn)
import numpy as np
from simtk.openmm.app import *
import simtk.openmm as mm
from simtk.unit import *
from simtk.openmm.app.internal.unitcell import computePeriodicBoxVectors
import json

def create_structure(atom_number=100):
    n  = atom_number
    d1 = 3.10
    d2 = 22.4
    x = np.zeros(n)
    y = np.arange(n) % 2 * 22.2 + np.random.normal(0,0.1,n)
    z = np.arange(n)*3.1 + np.random.normal(0,0.1,n)

    coor = np.vstack([x,y,z]).T
    return coor

def add(st, n):
    if len(st) > n:
        return st[:n]
    else:
        return st + " " * (n - len(st))

def save_pdb(coor):
    coor = np.asarray(coor)
    record = ""
    for i in np.arange(coor.shape[0]):
        ret = ""
        atomNum = i
        segmentNum = i
        line = coor[i]
        ret = add("ATOM", 6)
        ret = add(ret + "{:5d}".format(atomNum), 11)
        ret = ret + " "
        ret = add(ret + "CA", 17)
        ret = add(ret + "ALA", 21)
        ret = add(ret + "A", 22)
        ret = add(ret + str(atomNum), 26)
        ret = add(ret + "         ", 30)
        # ret = add(ret + "%i" % (atomNum), 30)
        ret = add(ret + ("%8.3f" % line[0]), 38)
        ret = add(ret + ("%8.3f" % line[1]), 46)
        ret = add(ret + ("%8.3f" % line[2]), 54)
        ret = add(ret + (" 1.00"), 61)
        ret = add(ret + str(float(i % 8 > 4)), 67)
```

```
        ret = add(ret, 73)
        ret = add(ret + str(segmentNum), 77)
        record += ret + "\n"

    return record

def create_system(atom_number=100):
    system = mm.System()
    for i in np.arange(atom_number):
        system.addParticle(181017) # The particle mass was choosen as the mass of a nucleosm
    # add bond force
    force = mm.HarmonicBondForce()
    force.setForceGroup(1)
    for i in np.arange(atom_number - 1):
        force.addBond(int(i),int(i+1),22.4,2 * 13.389)
    # link first and last atom
    force.addBond(0,int(atom_number - 1),22.4,2 * 13.389)
    force.addBond(0,int(atom_number - 2),6.2,2*5)
    force.addBond(1,int(atom_number - 1),6.2,2*5)
    force.setUsesPeriodicBoundaryConditions(True)
    system.addForce(force)
    # setup PBC
    a = computePeriodicBoxVectors(310,310,310,90*degrees,90*degrees,90*degrees)
    system.setDefaultPeriodicBoxVectors(*a)
    # add custombonded force
    morse_force = mm.CustomNonbondedForce("exp(-0.01213*r + 0.92086)")
    morse_force.setForceGroup(2)
    morse_force.setNonbondedMethod(morse_force.CutoffPeriodic)
    morse_force.setCutoffDistance(9)
    for i in np.arange(atom_number):
        morse_force.addParticle(())
    system.addForce(morse_force)

    if False:
        system.addForce(mm.CMMotionRemover())
    return system

if __name__ == "__main__":
    coor = create_structure(atom_number=100)
    system = create_system(atom_number=100)
    integrator = mm.VerletIntegrator(1*picoseconds) # The particle mass is very large. So 1

    simulation = Simulation(topology=None, system=system, integrator=integrator)
    simulation.context.setPositions(coor)
    energy = []
    position = []
    for i in range(1000):
        simulation.integrator.step(1000)
        # get position
```

```
        state = simulation.context.getState(getPositions=True, getEnergy=True, groups=4)
        positions = state.getPositions().value_in_unit(angstroms)
        position.append(positions)
    with open("position.json",'w') as f:
        json.dump(position, f)
    with open("pdb_notail.pdb",'w') as f:
        for i,v in enumerate(position):
            f.write("MODEL     %4d\n"%(i+1))
            f.write(save_pdb(v))
            f.write("ENDMDL\n")
```
